# MCU promotes the migration of glioma cells by activating p38 through TFEB-mediated autophagy

**DOI:** 10.7150/jca.89485

**Published:** 2024-01-12

**Authors:** Jialong Chen, Renjian Lu, Yongming Peng, Yixi Lai, ZiWei Cai, Dongyan Zheng, Ailan Xie, Kailun Huang, Congmin Liang, He Zhang

**Affiliations:** Dongguan Key Laboratory of Environmental Medicine, The First Dongguan Affiliated Hospital, School of Public Health, Guangdong Medical University, Dongguan, 523808.

**Keywords:** Autophagy, MCU, p38, Glioma, migration

## Abstract

Changes in calcium signalling are crucial for the development of glioma cells. Whether mitochondrial calcium balance is involved in glial cell development is still unknown. Mitochondrial Calcium Uniporter (MCU) plays an important role in regulating glioma progression. In this work, we found that MCU and p38 expression were positively correlated with glioma grade and the degree tumour progression. MCU increases glioma cell migration by upregulating p38. Furthermore, p38 promotes glioma progression by activating Transcription Factor EB (TFEB)-mediated autophagy. Thus, MCU promotes glioma cell migration by activating autophagy in a p38/TFEB pathway-dependent manner, which provides a theoretical basis for new therapeutic targets for gliomas.

## Introduction

Glioma is the most common primary malignant tumour of the central nervous system. According to the statistics of the American Brain Tumour Registry, glioma accounts for approximately 80% of primary malignant tumours of the central nervous system and can occur in any part of the central nervous system. Glioma has complex molecular genetic characteristics and is characterized by high incidence, high malignancy and a poor prognosis 1. According to the WHO classification of tumours of the central nervous system, gliomas are classified into four grades (I, II, III and IV), among which grade I and II gliomas are considered low-grade gliomas, while III and IV gliomas are high-grade gliomas 2; glioblastoma is the most common high-grade glioma, and it has the worst prognosis, with a median survival time of approximately 15 months 3. High-grade gliomas have biological characteristics such as rapid growth, abnormal vascular proliferation and easy destruction of brain tissue. Therefore, the treatment of malignant glioma is difficult, and the prognosis is poor 4.

The cause of glioma is not clear, and previous studies have revealed that the risk factors for glioma include genetic factors, ionizing radiation, chemical factors and viral infection 5. At present, the treatment of glioma by clinicians at home and abroad is mainly surgical treatment combined with comprehensive treatment such as radiotherapy and chemotherapy, but the treatment effect is still not satisfactory. Although some new biological therapies (immunotherapy) and gene therapy have facilitated progress, they are mostly limited to animal experiments or preliminary clinical trials 4. Therefore, it is important to further explore the pathogenesis and new therapeutic strategies of glioma and to explore new molecular therapeutic targets for glioma.

With the deepening of research on the molecular mechanism of tumour tissues, the relationship between mitochondrial calcium homeostasis and the development of malignant tumours has attracted much attention. MCU is the main mechanism by which calcium flows into mitochondria, and it controls cell energy metabolism and promotes reactive oxygen species production and programmed cell death 6. It is believed that the expression of MCU is related to tumour size and lymphatic infiltration, which suggests that it supports tumour growth and metastasis 7. More research shows that the increase in MCU expression levels affects the migration of glioma cells, which leads to the malignancy of glioma. In glioma cells, the lysosomal defect induced by quercetin and chloroquine can cause stress on the endoplasmic reticulum and mitochondria, thus causing functional defects and inducing glioma cell death 8. In addition, the relationship between high MCU levels and a high calcium transient value and malignant glioma was clarified. The abnormal increase in the calcium transient value can activate biochemical pathways involved in cell proliferation, which is the key sign of malignant gliomas. Due to the decrease in cell proliferation after low expression of MCU, targeting the MCU complex and intracellular calcium regulation may be a new unexplored strategy for the treatment of glioma 9. However, the regulatory mechanism of MCU as a therapeutic target of nervous system tumours needs to be further explored, and the mechanism of MCU's influence on glioma migration and its molecular mechanism deserves further study.

Previous studies have shown that the mitogen-activated protein kinase (MAPK) family plays an important role in carcinogenesis 10. The activation of the p38 MAPK signalling pathway is realized through a phosphorylation cascade 11, and the phosphorylation of p38 can regulate key proteins in autophagy and apoptosis, leading to cell death. In glioma cells, by binding with p38 and inhibiting the phosphorylation of the p38 protein, the p38 MAPK signalling pathway is inhibited, which proves that this pathway can downregulate the apoptosis and autophagy of glioma cells 12. In addition, studies have shown that inhibiting p38 phosphorylation negatively regulates the p38 MAPK pathway and inhibits the apoptosis of glioma cells through the p38 MAPK pathway, which negatively regulates the p38 MAPK pathway and leads to the survival of glioma cells. Therefore, p38 is considered an inducer of glioma cell apoptosis. However, Wang Xinyu et al. 14 showed that ruthenium red, an inhibitor of MCU, can inhibit rank-induced ROS production and NFATc1 activation through the p38 MAPK signalling pathway, thus alleviating the generation of osteoclasts and ovariectomized osteoporosis. Gulin et al. found 15 that MCU regulates the expression of PGC-1α and mediates the metabolic reprogramming of pulmonary fibrosis by increasing the phosphorylation of ATF-2 through p38 MAPK in a redox-dependent manner. Targeting MCU complexes to affect the migration of glioma by stacking p38 inhibitors may be a new future exploration strategy for the treatment of glioma. However, the regulatory mechanism involving MCU and p38 in the targeted treatment of cancer still needs further exploration. The impact mechanism and molecular mechanism of MCU activation of p38 on glioma migration deserves further exploration and research.

Autophagy is a process of cell self-digestion that can engulf damaged, misfolded and nonfunctional proteins and degrade them through lysosomes. TFEB is one of the most important transcription factors found in recent years to regulate autophagosome pathway. TFEB is the main gene that regulates lysosomal biogenesis. It encodes a transcription factor, which can increase autophagy pathway and lysosomal function by promoting the expression of several autophagy-related genes (such as LC3Ⅱand Beclin-1) and lysosomal-related genes (such as LAMP1 and CTSD), thus promoting the degradation and removal of intracellular wastes. Autophagy occurs in different stages of tumour progression. In recent years, increasing *in vivo* and *in vitro* experimental evidence has shown that autophagy plays an important role in glioma 16. For example, Jiang Tao and his colleagues found that compared with low-grade glioma tissues, Lc3B and p62 were highly expressed in glioma tissues of advanced patients, and the expression of Lc3B and p62 was positively correlated with tumour grade, patient-free survival and overall survival 17. However, another researcher reported that patients with low expression of Lc3B/beclin1 had better progression-free survival 18. These results suggest that autophagy may play different roles in different patients or tissues of different tumour stages. In addition, studies have shown that autophagy and apoptosis of glioma cells can be inhibited by inhibiting p38 MAPK signalling. In contrast, when p38 MAPK signal activity is enhanced, and autophagy and apoptosis of glioma cells are promoted. Inhibition of p38 phosphorylation can suppress autophagy and apoptosis in glioma cells 11. Therefore, it is of great significance to regulate autophagy through p38 to influence the migration of glioma cells.

In this study, we found that the migration of Human malignant glioma (U87) cells was affected by spermine and ruthenium red treatment, and the expression of the migration protein Vimentin was affected, which confirmed that MCU could affect the migration of glioma cells. In addition, the mechanism by which MCU regulates autophagy in glioma cell migration was clarified, providing a new theoretical basis and molecular therapeutic target for further understanding the influence of MCU on glioma.

## Materials and methods

### Data mining from public databases

The online website Cancer Cell Line Encyclopedia (CCLE, https://portals.broadinstitute.org/ccle), The Human Protein Atlas (https://www.proteinatlas.org/) and Gene Expression Profiling Interactive Analysis (GEPIA, http://gepia.cancer-pku.cn/index.html) were used to investigate the expression of MCU, p38 and TFEB. Then, the correlation between MCU, p38 and TFEB in different stages of glioma was determined via the online website GEPIA and Chinese Glioma Genome Atlas (CGGA, http://www.cgga.org.cn/index.jsp).

### Cell cultures and treatments

U87 human Glioblastoma multiforme (GBM) cells were cultured in Dulbecco's Modified Eagle Medium (DMEM) supplemented with 10% (Foetal Bovine Serum) FBS (Sangon Biotech; Uruguay, South America) and penicillin/streptomycin (Beyotime, China) at 37 °C in a humidified atmosphere of 5% CO2. Cells were treated with the following agents for 24 h: 10 µM ruthenium red (RR), 10 µM spermine (SP), 20 µM chloroquine (CQ), 20 µM rapamycin (RAPA), and 10 µM SB 202190 (SB). The culture dish was purchased from Sorfa (Beijing, China).

### Cell transfection and plasmids

Cell transfection was achieved by using Lipofectamine 3000 reagent (Invitrogen, Carlsbad, CA, USA) for plasmid and siRNA following the manufacturer's protocols. The culture dish was purchased from Sorfa (Beijing, China). The overexpressed MCU plasmid (VSVG-pLKD-U6-hMCU plasmid) was constructed by Lucky Biotechnology Corporation (Shanghai, China).

### Migration assay

Wound healing assay: Constructed cells were seeded in a 24-well culture plate (1 × 10^5^/well) and placed at 37 °C and 5% CO2 for 24 h. The medium was removed, and the surface of the inoculated cells was scratched with a 10 μl pipette tip and marked. The samples were washed gently twice with Phosphate Buffered Solution (PBS), and 1 ml of medium containing 10% serum was added. The scratches were photographed at 0 h and 24 h. The experiment was conducted with three replicates and repeated five times. The distance that the cells migrated into the wounded area during this time was measured. The results are expressed as the migration index (the migration distance of cells in the experimental group relative to the migration distance of cells in the NC group).

Transwell cell migration assay: Constructed cells were seeded in wells of Matrigel plates containing serum-free and high-glucose DMEM (1 × 10^5^ cells/well). The lower well contained 500 µl of complete medium (DMEM and 10% FBS). After incubation at 37 °C for 48 h, cells that had not migrated through the well were gently removed with a cotton swab. The cells in the lower chamber were fixed with 5% glutaraldehyde for 10 min, stained with 1% crystal violet in 2% ethanol at room temperature for 20 min, photographed and counted.

### Western blotting (WB)

Cells were lysed in Radio-Immunoprecipitation Assay (RIPA) buffer (Beyotime, Shanghai, China) containing Phenylmethylsulfonyl fluoride (PMSF) (dilution, 1:100; Beyotime, Shanghai, China). Then, the protein was collected by centrifugation and boiled at 100 ℃ for 5 minutes after adding loading buffer (1:4; Beyotime, Shanghai, China). The concentration of the proteins was measured by using a Bicinchoninic Acid Assay (BCA) Protein Assay Kit (Beyotime, Shanghai, China) after total proteins were extracted with the use of a cell lysis buffer that was purchased from KeyGEN (KGP701, Nanjing, China). The collected proteins were incubated overnight at 4 °C with primary antibody and then cultured with agar beads for 3 hours on the second day. Twenty micrograms of extracted protein from each sample was separated by sodium dodecyl sulfate-polyacrylamide gel electrophoresis (SDS‐PAGE) electrophoresis and transferred to polyvinylidene fluoride (PVDF) membranes. After blocking for 1 h in 5% bovine serum albumin (BSA), the membrane was incubated overnight at 4 °C with primary antibodies, including rabbit anti-MCU (1:1500, Cell Signaling), mouse anti-P38 (1:1500, Cell Signaling), anti-rabbit phospho-P38 (Thr180/Tyr182) (1:1500, Cell Signaling), rabbit anti-TFEB (1:1000, Proteintech), rabbit anti-LC3 (1:1000, Proteintech), mouse anti-vimentin (1:1000, Santa Cruz), mouse anti-tubulin (1:1000, Proteintech) and mouse anti-GAPDH (1:1000, Beyotime). Then, the membranes were incubated with the corresponding horseradish peroxidase (HRP)-conjugated secondary antibody (1:1000) at room temperature for 60 minutes. Finally, the protein-antibody complexes were visualized by an enhanced chemiluminescence detection system and analysed with ImageJ software.

### Immunofluorescence (IF) assay

Cells were washed twice in PBS, fixed in 4% paraformaldehyde for 15 min and permeabilized with precooled methanol for 10 min. Then, the cells were incubated in 5% BSA blocking buffer for 30 min. The cells were incubated with rabbit anti-TFEB (1:100, Proteintech) at 4 °C overnight, incubated the next day with anti-rabbit CoraLite 594-conjugated (1:100, Proteintech) secondary antibody for 2 h at room temperature, and washed three times; DAPI was added, and the samples were blocked with glycerol and observed and photographed under a confocal microscope.

### Immunohistochemistry (IHC)

Surgically resected glioma tissues were harvested from 15 patients (grade II: five cases, grade III: five cases, grade IV: five cases) at Shenzhen People's Hospital, China, after obtaining informed consent from the patients and in line with the guidelines of the Research Ethics Committee.Inclusion criteria of glioma population samples: ①age, ②pathological grade, ③molecular markers, ④functional status, and ⑤living habits. All samples were preserved in formalin or expeditiously frozen and stored at -80 °C after surgery. Paraffin-embedded tissue sections were deparaffinized with xylene, and endogenous peroxidase activity was quenched with 3% H_2_O_2_ in methanol for 25 minutes in the dark. Tissue sections were dehydrated through graded alcohols and subjected to antigen retrieval using sodium citrate. After washing with PBS three times and blocking with 3% BSA for 30 minutes, the sections were incubated sequentially with primary antibodies at 4 °C overnight. The next day, sections were then washed for 5 minutes in PBS and incubated for 1 hour with the respective secondary antibody (Servicebio, Wuhan, China). After washing, sections were incubated with DAB (3,3′-diaminobenzidine tetrahydrochloride) (Servicebio, Wuhan, China) and immediately washed under tap water after colour development. Sections were then counterstained with haematoxylin. Slides were mounted with DPX (dibutyl phthalate xylene) and were then observed under a light microscope.

### Statistical analyses

All data are presented as the mean ± standard deviation (SD) from at least 3 independent experiments. Unpaired/paired Student's t tests were used to identify statistically significant differences between two groups, and one-way ANOVA followed by Dunnett's multiple comparisons tests was used to identify statistically significant differences between more than two groups. All statistical analyses were performed using GraphPad Prism version 8 (GraphPad Inc., La Jolla, CA, USA). Differences with *p* values < 0.05 were considered statistically significant.

## Results

### MCU promotes tumour progression-related behaviours in glioma cells

**Figure 1:**
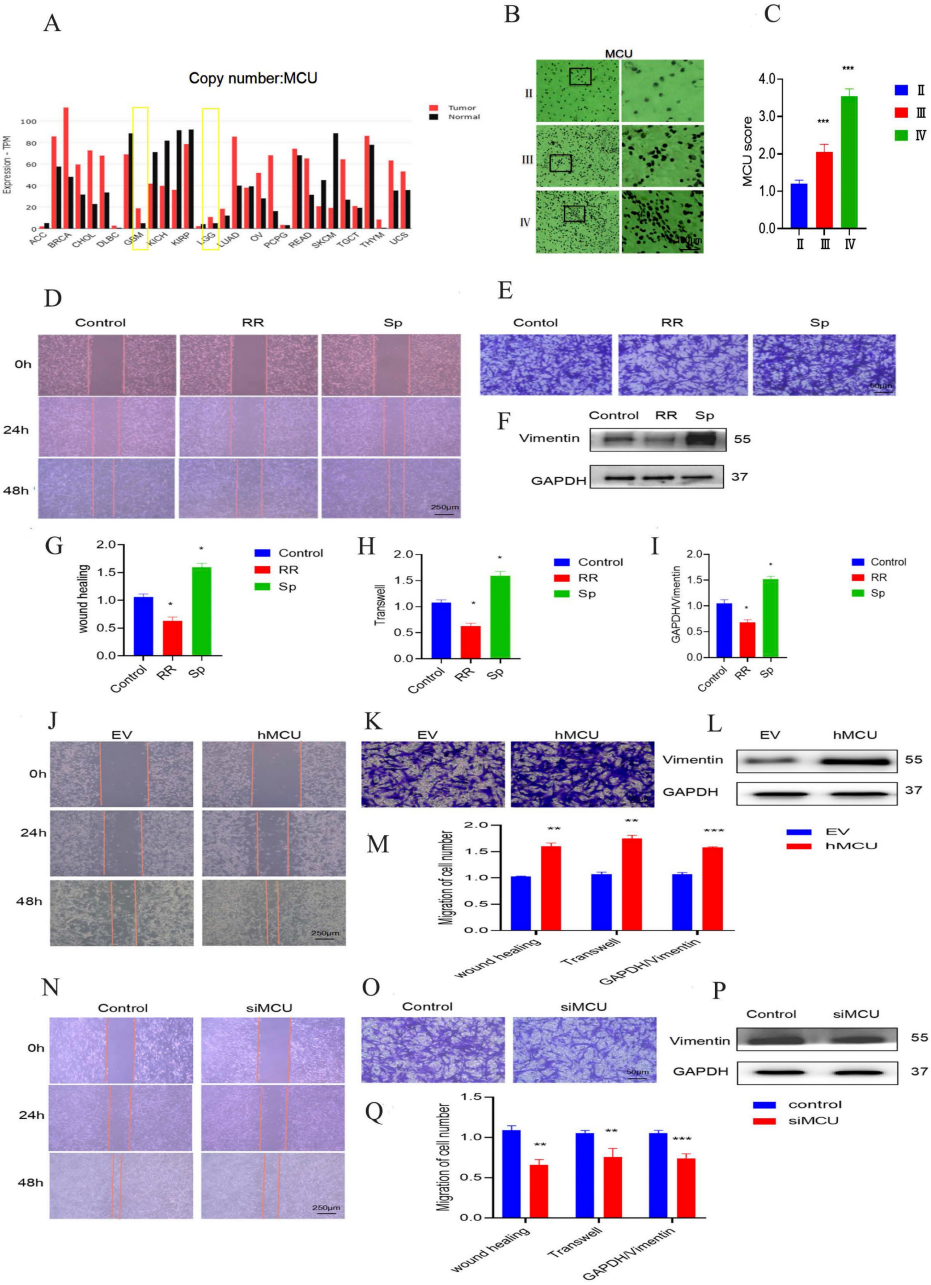
** The effect of MCU on glioma cell migration.** A):CGGA online website search found that MCU expression was significantly higher in GBM than in LGG; B, C):IHC results of human glioma samples showed a gradual enhancement of MCU expression in glioma from grade to grade (ruler 100 μm); D, E, G, H): The cell scratch experiment (ruler 250μm) and transwell experiment (ruler 50μm) of U87 cells treated by RR and Sp analyzed the changes of cell migration function and the corresponding results; F, I): WB measured the expression results and analysis of migration protein Vimentin in U87 cells treated with RR and Sp; J, K,M): Cell scratch experiment (ruler 250μm) and transwell migration experiment (ruler 50μm) detected the changes of siMCU and the corresponding results; L,M): WB detected the expression of migration protein Vimentin in U87 cells treated with siMCU; N,O,Q): Cell scratch assay (ruler 250 μm) and transwell migration assay (ruler 50μm) detected the migration of hMCU to U87 cells; P,Q): WB measured the expression of migration protein Vimentin in U87 cells treated with hMCU. All the results are expressed as mean ± SD (*P < 0.05, **P < 0.01, ***P < 0.001).

Through the analysis of the China Glioma Genome Atlas (CGGA) database, it was found that there is a certain positive correlation between the expression of MCU and the expression of glioma cell markers (Figure 1A), suggesting that MCU may be highly expressed in glioma cells. Furthermore, the levels of MCU expression in gliomas from grade II to IV were examined by IHC. As shown in Figure 1 B and C, the expression of MCU increased gradually from grade II to grade IV glioma. To verify the role of MCU in the development of glioma, wound healing and transwell assays were performed to elucidate the effect of MCU on the migration of U87 cells. We compared normal Human astrocyte (NHA) with Human glioma cells (U251), Astrocytoma cells of human brain (U118) and U87, and WB detected related proteins. The results are shown in S1. Finally, we chose U87 as the experimental cell *in vitro*. Compared to control cells, the number of migratory U87 cells decreased at 48 h in response to treatment with RR and SP (Figure 1D,E,G,H). The experimental results of WB to detect the level of Vimentin, which is functionally involved in migration, were consistent with the above results (Figure 1F,I).

Furthermore, we treated U87 cells with siMCU or VSVG-pLKD-U6-hMCU. The results of the wound healing and transwell experiments of U87 cells treated with siMCU led to a marked decrease in cell migration (Figure 1J-M), while the migration ability of U87 cells treated with siMCU and VSVG-pLKD-U6-hMCU was significantly enhanced (Figure 1N-Q). Taken together, these findings indicate that as a potent regulator, MCU facilitates the migration of glioma cells.

### MCU regulates the migration of glioma cells by activating p38

These results of Figure 1 confirm that MCU plays a pivotal role in glioma migration, but the potential mechanisms remain to be further revealed. Therefore, the GEPIA web server (http://gepia.cancer-pku.cn/detail.php?gene=p38) was used to investigate the underlying mechanisms.

**Figure 2:**
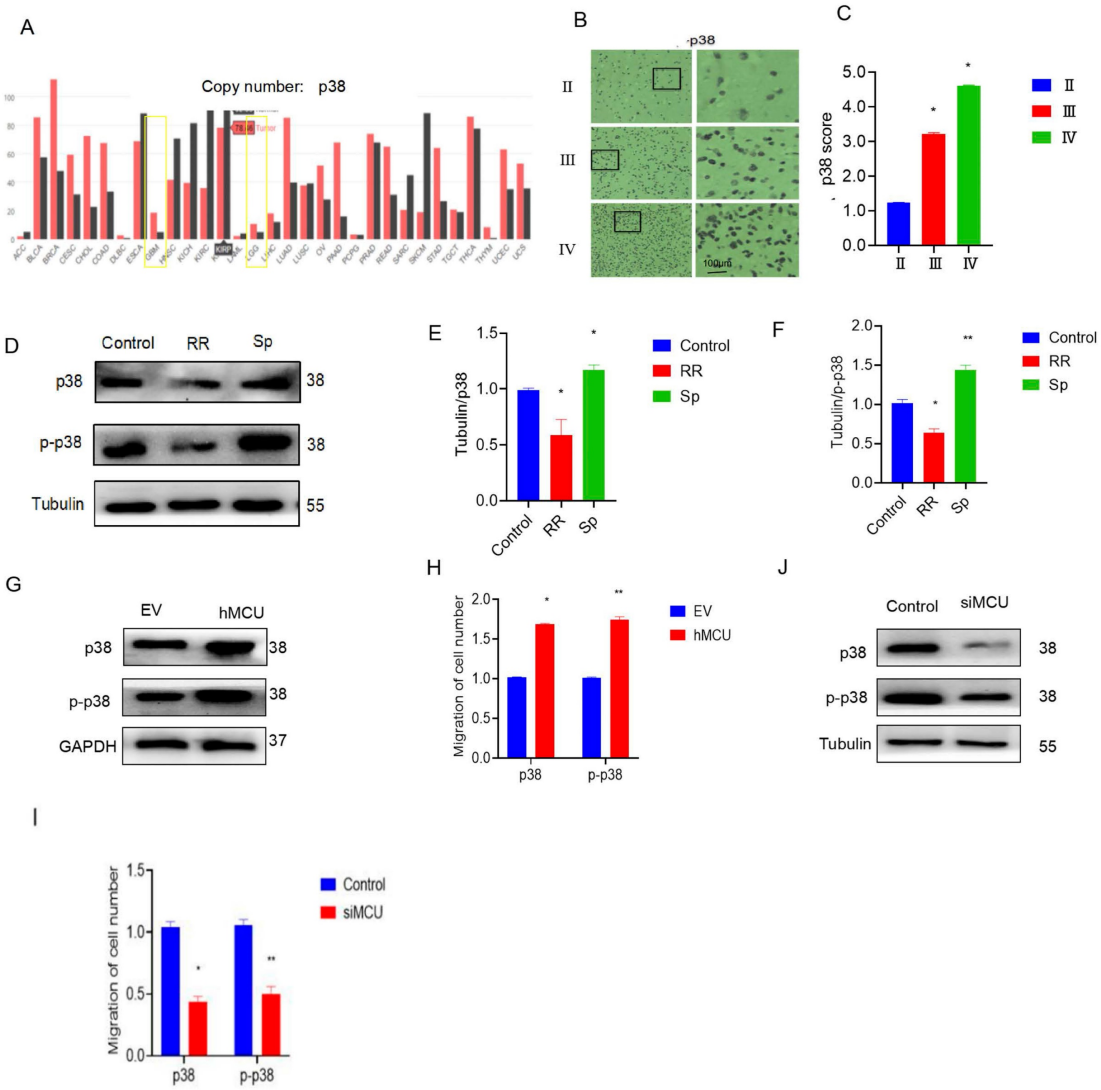
** MCU regulated p38 expression in glioma cells.** A): CGGA online search found p38 expression in LGG, GBM; B-C): p38 expression in human glioma specimens by immunohistochemistry (ruler 100μm; D-F): p38 and p-p38 protein expression after RR or Sp treated U87 cells; G-I): U87 cells with siMCU and hMCU: WB experiment to detect p38 and p-p38 protein expression; EV was empty plasmid control group, results in mean ± SD (*P < 0.05, **P < 0.01).

We found that the expression of p38 in LGG and GBM tissues was much higher than that in normal tissues (Figure 2A). Immunohistochemical results also showed that the expression of p38 in human glioma specimens increased with increasing malignant degree of glioma (Figure 2B,C). To verify the regulatory effect of MCU on p38, an MCU agonist or inhibitor was added to U87 cells. WB experiments showed that RR could reduce p38 and p-p38 protein levels, while Sp could increase the protein levels of p38 and p-p38 (Figure 2D-F). To further clarify the regulation of MCU on p38, specific siRNA interference was used to construct U87 cells with low MCU expression, and the VSVG-pLKD-U6-hMCU plasmid was used to construct U87 cells with high MCU expression. WB experiments showed that overexpression of MCU increased the levels of p38 and p-p38 proteins (Figure 2G,H), while knockdown of MCU reduced the levels of p38 and p-p38 proteins (Figure 2J,I). The above results suggest that p38 activation is positively related to glioma malignancy and that MCU can regulate the expression of p38. The above results suggest that p38 activation has a positive correlation with glioma malignancy and that MCU can regulate the expression of p38.

**Figure 3:**
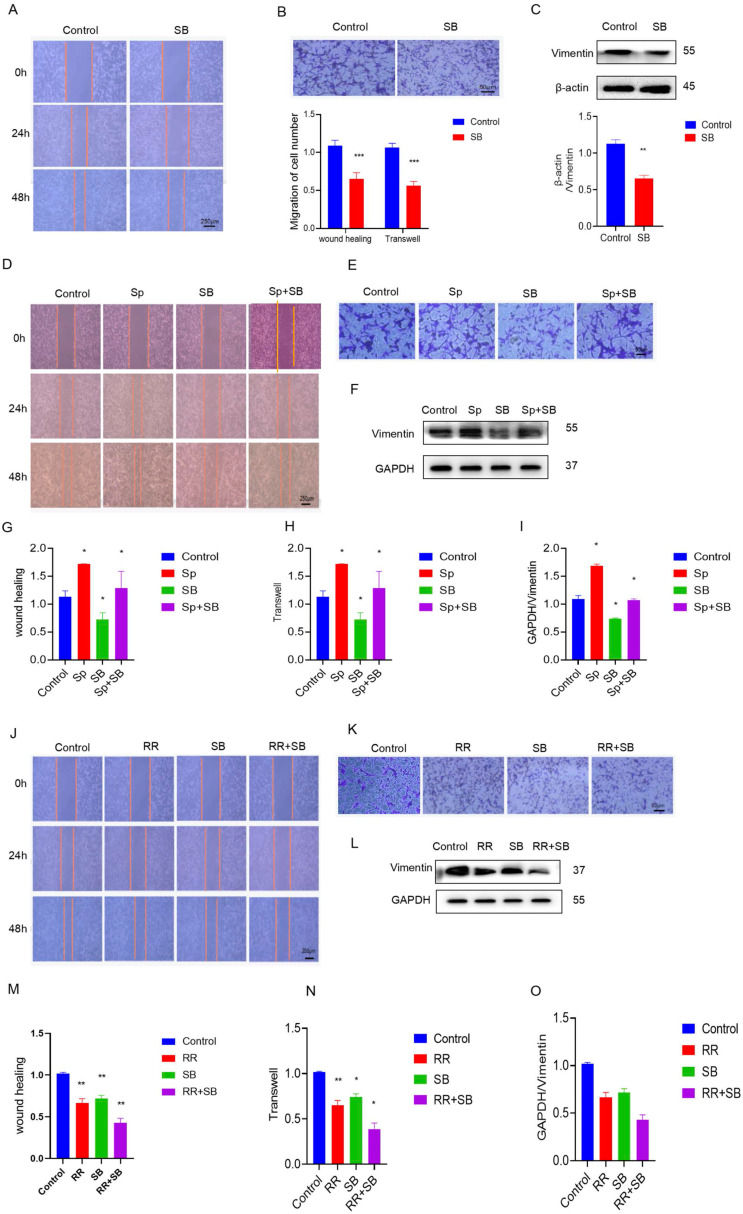
** MCU promotes glioma cell migration by p38.** A, B): SB treated the cell scratch experiment (ruler 250μm) and transwell experiment (ruler 50 μm) to detect the changes in migration ability and analyze the corresponding results; C): expression of migration protein Vimentin was detected by WB after treatment of U87 cells with SB; D-I): After adding SB and Sp, the cell scratch experiment (ruler 250μm) and transwell experiment (ruler 50μm) were used to detect the change in migration ability and WB detected the expression results of the migration protein Vimentin and the corresponding analysis; J-O): After adding SB and RR, the cell scratch experiment (ruler 250μm) and transwell experiment (ruler 50μm) were used to detect the change in migration ability and WB detected the expression results of the migration protein Vimentin and the corresponding analysis,the results are expressed as mean ± SD (* P <0.05, * * P <0.01).

To determine the effect of MCU on glioma cell migration through p38, the migration ability of U87 cells was evaluated. The addition of SB, SP, and RR reduced cell migration ability, as shown by the wound healing assay and transwell assay (Figure 3A-I). In addition, compared with that of siMCU-U87 cells, the migration of MCU-overexpressing U87 cells was reduced after SB treatment (Figure 3J-O). Similarly, WB analysis showed that Vimentin expression was decreased after siMCU treatment of U87 cells, and the same changes occurred after SB was used to treat U87 cells transfected with siMCU (Figure S2A-L). In summary, MCU can affect the migration of glioma cells through p38.

### MCU affects the migration of glioma cells through the regulation of autophagy by p38

**Figure 5:**
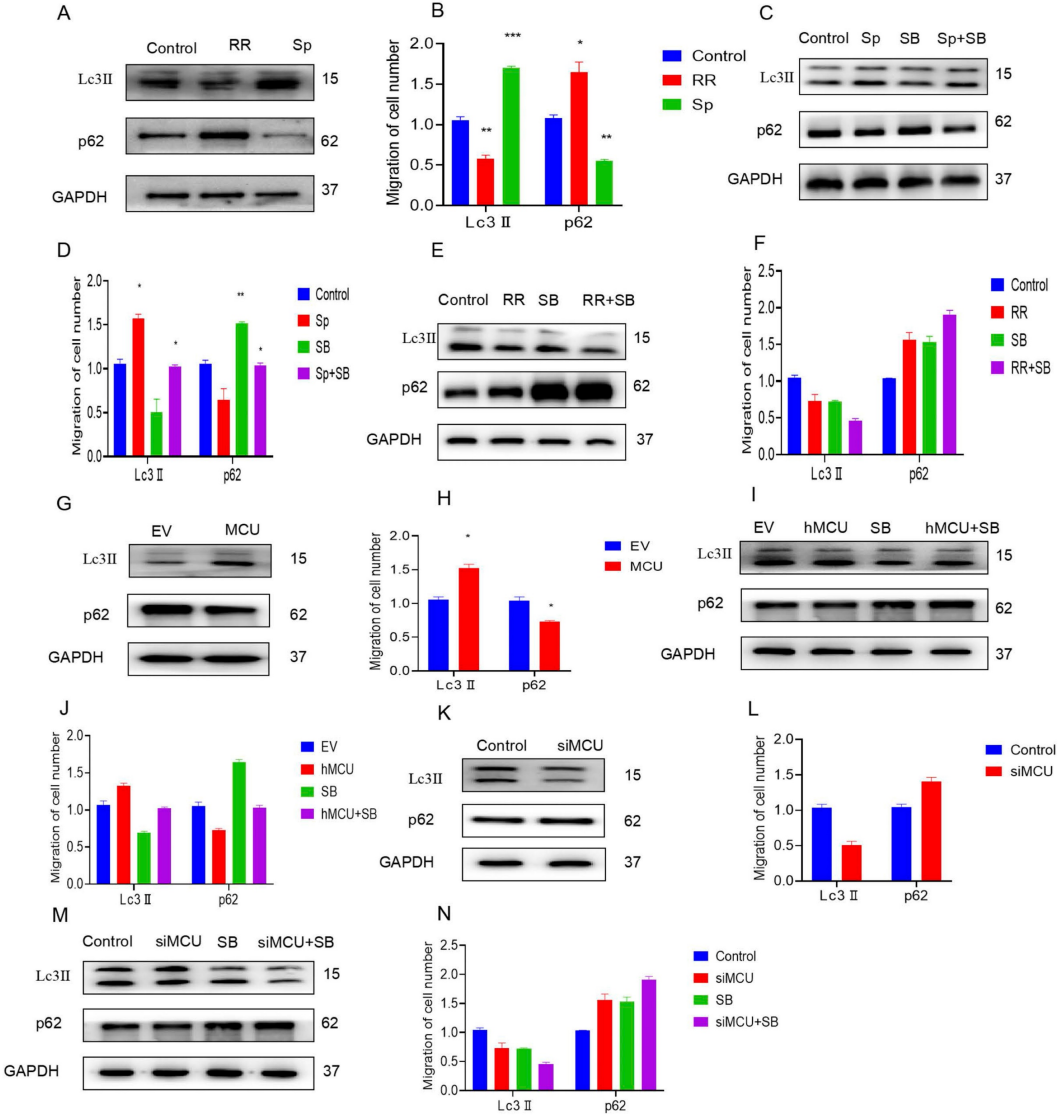
** MCU regulates autophagy by p38.** A, B): U87 cells were treated with RR and Sp, and the expression levels of Lc3Ⅱand p62 autophagy were determined by WB; C-F): After SB was added, WB experiment was carried out to detect the autophagy-related expression levels of Lc3Ⅱand p62.; G, H, K, L):U87 cells that overexpressed and interfered with MCU were constructed by molecular biology method, and the autophagy-related expression levels of Lc3Ⅱand p62 were detected by WB experiment.; I, J, M, N): The molecular biological method was used to construct U87 cells that overexpressed MCU and interfered with MCU, and then SB was added. WB experiment was used to detect the autophagy-related expression levels of Lc3Ⅱand p62, results are expressed as mean ± SD (*P < 0.05, **P < 0.01, ***P < 0.001).

Previous studies have found that autophagy participates in the development process of glioma and that glioblastoma cells suppress astrocyte transformation through macroautophagy. Immunoblot results of human glioma and stage tissue samples showed that as the expression of Lc3 increased, the expression of p62 also decreased (Figure 5A,B). TFEB is an important transcription factor mediating autophagy. According to the online website of the China Glioma Genome Atlas Project, the expression of TFEB in GBM was significantly higher than that in LGG (Figure 5C). Immunoblotting and immunohistochemistry of human glioma samples of different stages also showed that the expression of TFEB in human glioma specimens increased as the degree of glioma malignancy increased (Figure 5D-F).

To verify the role of autophagy in glioma migration, U87 cells were used as a model, and the effect of autophagy on U87 cell migration was clarified through cell scratch tests and transwell experiments. The experimental results showed that chloroquine, an autophagy inhibitor, inhibited the migration of glioma cells, while rapamycin, an autophagy agonist, promoted the migration of glioma cells (Figures 5G, H, J, K). The WB results showed that CQ inhibited the expression of migration-related proteins, while RAPA promoted the expression of migration-related proteins (Figure 5I,L). The above results suggest that autophagy is involved in the regulation of glioma migration.

Then, by processing U87 cells and adding CQ and Sp, we found that CQ weakened Sp to promote the migration of U87 cells into the wound area, and we added CQ and RR to treat U87 cells. In the cell scratch experiment, the migration of U87 cells was significantly weakened (Figure 5M,N). With MCU knockdown and CQ treatment, the migration of U87 cells was significantly reduced in the cell scratch experiment, and the overexpression of MCU and CQ promoted the migration of MCU (Figure 5O, P). The above results suggest that MCU regulates glioma cell migration through autophagy.

**Figure 6:**
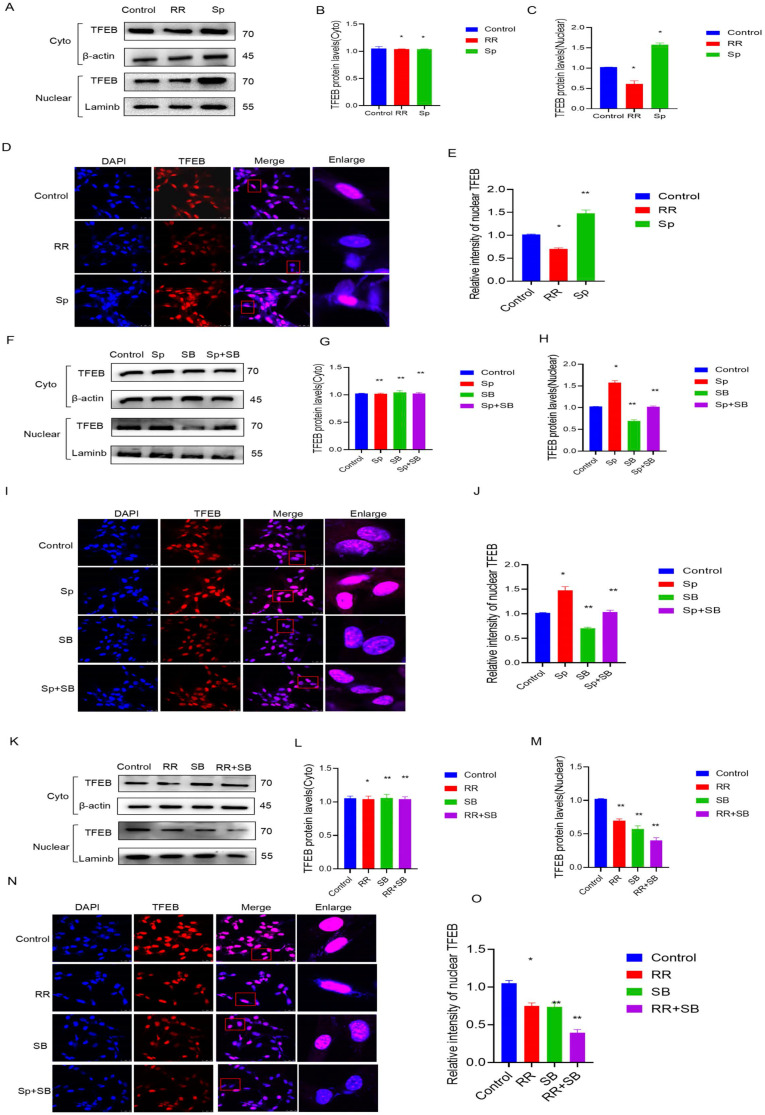
** MCU regulates TFEB-mediated autophagy by p38.** A-C): After RR and Sp were added, WB detected TFEB levels in cytoplasm and nucleus; D,E): IF detected the nuclear translocation level of TFEB after adding RR and Sp;F-H): After adding Sp and SB, WB detected the TFEB level in cytoplasm and nucleus; I-J): After adding Sp and SB, IF detected the nuclear translocation level of TFEB; K-M): After RR and SB were added, WB detected the TFEB level in cytoplasm and nucleus; N-O): IF detected the nuclear translocation level of TFEB after adding RR and SB. results are expressed as mean ± SD (*P < 0.05, **P < 0.01).

To explore the mechanism by which MCU regulates autophagy by activating p38, U87 cells were first treated with RR and Sp. The WB results showed that RR inhibited the expression of Lc3-II and increased the expression of p62. However, Sp increased the expression of Lc3-II and inhibited the expression of p62, suggesting that MCU regulates autophagy (Figure 6A,B). To further verify that MCU regulates autophagy by activating p38, RR and Sp were added to SB-treated U87 cells. WB results showed that Sp activated autophagy and that SB inhibited Sp-mediated regulation of autophagy (Figure 6C,D). The regulation of autophagy was obviously weakened by cotreatment with RR and SB (Figure 6E, F). Second, specific cells were constructed with U87 cells as the parental cells; the expression of LC3-II in hMCU increased and the expression of p62 decreased (Figure 6G,H). However, when the expression of LC3-II in siMCU decreased, the expression of p62 increased, which verified again that MCU regulates autophagy (Figure 6H,L). After the above treatment, SB was added, and it was found that SB attenuated autophagy induced by the MCU overexpression (Figure 6I,J), and autophagy induced by SB and MCU knockdown was obviously attenuated under dual treatment (Figure 6M,N). Thus, it was verified again that MCU can regulate autophagy through p38.

### MCU regulates TFEB-mediated autophagy through p38

Previous studies have shown that p38 is involved in the regulation of autophagic lysosomes mediated by TFEB, an important transcription factor. First, U87 cells were treated with RR and Sp, and it was found that the protein content of TFEB in the nucleus was decreased upon RR treatment, while the protein content of TFEB in the nucleus increased upon Sp treatment. Immunofluorescence experiments also showed that level of TFEB in the cytoplasm (indicating translocation) was decreased in cells treated with RR, while it was increased in cells treated with Sp (Figure 6A-E). The protein level of TFEB in the nucleus was reduced after adding SB, and the protein level of TFEB in the nucleus was significantly reduced by cotreatment with RR and SB. However, the protein content of Sp in the nucleus TFEB was reduced by SB treatment. Immunofluorescence experiments also confirmed this result (Figure 6F-O).

Plasmids were constructed to upregulate MCU expression in U87 cells, and specific siRNA was applied to interfere with MCU expression. The protein level of TFEB was decreased in cells treated with siMCU, and the protein level of TFEB was increased in cells treated with hMCU. The immunofluorescence experiment also confirmed this result (Figure S3A-C), (Figure S3F-H). The protein levels of TFEB in the cell nucleus were significantly reduced after SB and siMCU cotreatment. The increase in the protein level of TFEB in the nucleus induced by hMCU was attenuated by SB treatment. The immunofluorescence experiment also confirmed this result (Figure S3D, E, I, J). The above experimental results indicate that MCU regulates TFEB-mediated autophagy through p38.

## Discussion

An increasing number of studies have shown that MCU is closely related to cancer progression, and its potential mechanism differs significantly according to the types and stages of cancer. Many studies have shown that an increase in MCU expression is related to different types of cancer 19. For example, the expression of MCU is increased in various types of cancers, including breast cancer 8 and hepatocellular carcinoma 20. However, thus far, the biological function of MCU in glioma is not clear. It is more meaningful to study the molecular mechanism of MCU in glioma. In our research, we obtained three main results. First, we proved that MCU can promote the migration of glioma cells. Second, we found that MCU can promote the migration of glioma cells by activating p38. Third, we discussed that autophagy can promote the migration of glioma cells by activating p38 to regulate TFEB-mediated autophagy. First, in a bioinformatics search and immunohistochemical analysis of glioma samples, the expression of MCU increased with the malignant degree of glioma. Second, cell experiments demonstrated that knockdown of MCU and inhibitors of MCU significantly inhibit glioma cell migration, and overexpression of MCU and agonists of MCU significantly promote glioma cell migration. This proves that MCU plays an important role in regulating the migration of glioma cells, providing a new perspective.

As a key player in the MAPK family, the p38 mitogen-activated protein kinase (MAPK) signalling pathway plays a crucial role in a multitude of intracellular reactions, including inflammation, cell cycle regulation, cell death, development, differentiation, ageing, and tumorigenesis 9,11,21. In recent years, an increasing number of studies have focused on the effects of the p38 MAPK signalling pathway on glioma, a type of brain tumor. Studies have shown that the regulation of the p38 MAPK signalling pathway is closely associated with the proliferation, metastasis, and invasion of glioma. Previous research has also demonstrated that the activation of the p38 MAPK pathway can enhance the invasion of glioma 22. Therefore, based on relevant literature and the above experimental results, we further investigated whether there is a regulatory relationship between p38 and MCU in glioma migration.

To address this question, we first employed bioinformatics analysis and immunohistochemical staining of human glioma samples. Our analysis revealed that MCU and p38 were significantly overexpressed in glioma tissues compared to normal brain tissue. These data suggest a potential role for MCU and p38 in glioma development and progresssion. Subsequently, we employed pharmacological and molecular biological methods to treat cells and further investigate the relationship between MCU and p38. Our experiments demonstrated that the knockdown of MCU or the addition of inhibitors of MCU decreased the expression of p38 and phosphorylated p38 (p-p38), while the overexpression of MCU or the addition of agonists of MCU increased the expression of p38 and p-p38. These data suggest that MCU may regulate the expression and activation of p38 in glioma cells. To explore the functional consequences of this regulatory relationship, we performed migration-related experiments. Our results showed that inhibitors of p38 also inhibited the migration of glioma cells. Furthermore, when we treated cells with both MCU and the p38 inhibitor, our data revealed that MCU promoted glioma cell migration through the regulation of p38. Collectively, our findings suggest that MCU regulates glioma cell migration through modulation of the p38 MAPK signalling pathway.

Autophagy plays an important role in the occurrence and development of malignant tumours, so autophagy pathway-related genes or proteins are likely to be used as markers of glioma occurrence and prognosis. In recent years, many scholars have made continuous efforts to confirm this phenomenon. For example, Jiang Tao and his colleagues found that the expression of LC3Ⅱ and p62 was positively correlated with the tumour grade, free-moving survival time and overall survival time of tumour patients 17. In our study, we also found that autophagy activation can promote the migration of glioma cells, while autophagy inhibition can inhibit the migration of glioma cells. As an important transcription factor for autolysosomes, TFEB regulates the expression of lysosomal hydrolases, membrane proteins and genes involved in autophagy 23. TFEB is not only involved in the regulation of autophagosomes but can also be used as a target for many diseases, such as cancer. Studies have proven that an increase in TFEB activity can improve metabolism and promote the occurrence of cancer 24. In the advanced stage of tumour progression, autophagy can promote the dissemination of malignant tumour cells into the circulatory system, enhance cell colonization in other sites, organs or tissues, induce cells to enter dormancy and survive in the new environment, and increase cellular autophagy levels during cancer metastasis 25. It has been confirmed that the autophagy level is increased in the metastasis of breast cancer, melanoma, hepatocellular carcinoma and glioblastoma 26,27. However, in a bioinformatics search and immunohistochemical analysis of human glioma samples, the autophagy level and TFEB increased with the malignant degree of glioma. According to the experimental data, it is confirmed that the p38 MAPK inhibitor is an important gene for TFEB to promote the occurrence and development of autophagosomes. We also proved that p38 is the substrate of MCU and affects TFEB's function in regulating autophagosomes through its inhibition. The data show that a p38 inhibitor can affect TFEB function and then affect the migration of glioma cells. The above research discussed a new mechanism of MCU mediated by TFEB in glioma; that is, MCU regulates the expression of p38, inhibits the activity of p38, reduces the expression of TFEB, inhibits the migration of glioma cells, activates the activity of p38 and then increases the expression of TFEB. Finally, it promotes the migration of glioma cells.

As scientific research continues to advance, it has been found that the role of MCU and its regulators in tumour is becoming increasingly clear. MCU is a key regulator of mitochondrial calcium uptake, which plays an important role in cell signalling, energy production, and cell death. However, the specific mechanism of MCU in tumour development and progression is not fully understood. Therefore, further research is needed to explore the role of MCU in cancer pathobiology and to provide a theoretical basis for tumour treatment. In order to accurately evaluate the role of MCU in tumour, it is necessary to comprehensively study the effects of MCU on proliferation, migration, invasion, adhesion, starvation or various drug treatments in multiple cell lines. Experimental data have shown that MCU affects the migration of glioma cells. When MCU is knocked down or inhibited, the migration of glioma cells is specifically inhibited. On the contrary, when MCU is overexpressed or agonist-treated, the migration of glioma cells is promoted. This suggests that MCU may be a potential target for glioma treatment. In addition, animal experiments have shown that MCU affects the tumour formation capacity of nude mice and affects the development of glioma. Therefore, it can be speculated that targeting MCU may also have potential therapeutic value for other types of tumours.

In summary, our results show that MCU regulates TFEB-mediated autophagy and promotes the migration of glioma cells through p38. The results provide a new perspective on the role of mitochondrial calcium transporters and p38 in autophagy and glioma. Notably, inhibiting the expression of MCU can weaken the function of autophagosomes and inhibit the migration of glioma cells to prevent the progression of glioma. The experimental results can provide potential strategies for the treatment of glioma. This research is limited to the preliminary discussion of this phenomenon. The signalling pathway and mechanism involved in the occurrence and development of glioma are very complicated, and there are also complex and close relationships among autophagy, p38MAPK and MCU. Therefore, the exact mechanism of action between them and the role of the p38MAPK signalling pathway need to be further explored.

## Supplementary Material

Supplementary figures.

## Figures and Tables

**Figure 4:**
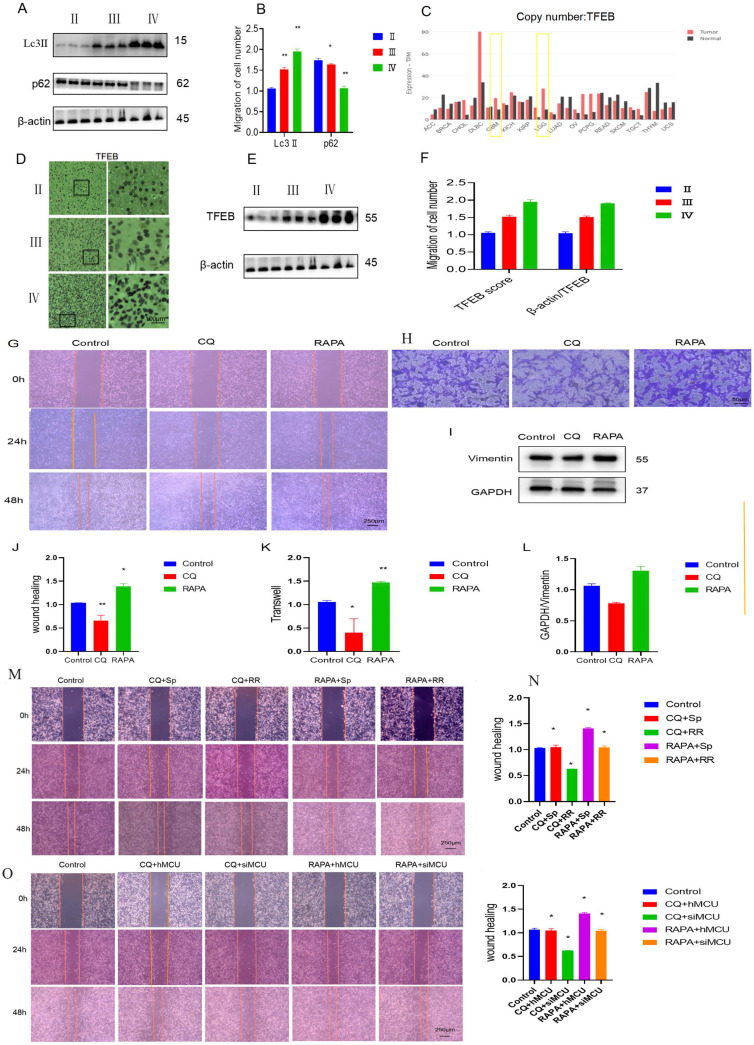
** MCU regulates the migration of glioma by autophagy**. A,B): WB detection of autophagy-related expression levels of Lc3Ⅱand p62 in human glioma samples.; C): CGGA website search found that TFEB expression in GBM was significantly higher than that in LGG.; D-F): The results of WB and IHC showed that the expression of TFEB in human glioma specimens increased with the increase of malignant degree of glioma (ruler 100 μm).; G, H, J, K): After U87 cells were treated with CQ and RAPA, cell scratch test (ruler 250μm) and transwell test (ruler 50μm) were carried out to detect the change of migration ability.; I, L): WB to detect the expression of migration protein Vimentin; M,N):After U87 cells were treated with CQ and RAPA,Sp and RAPA,CQ and Sp, CQ and RRcell scratch test (ruler 250μm) and transwell test (ruler 50μm) were carried out to detect the change of migration ability;O,P):After U87 cells were treated with CQ and hMCU,CQand siMCU, RAPA and hMCU, RAPAand siMCU cell scratch test (ruler 250μm) and transwell test (ruler 50μm) were carried out to detect the change of migration ability, the results are expressed as mean ± SD (*P < 0.05, **P < 0.01).
