# Supplementary material for: MCU promotes the migration of glioma cells by activating p38 through TFEB-mediated autophagy
Source: J Cancer. 2024 Jan 12;15(5):1257–70. doi: 10.7150/jca.89485 (PMC10861810; doi:10.7150/jca.89485)
Supplement: Supplementary file 1 — Supplementary figures. [file jcav15p1257s1.pdf]

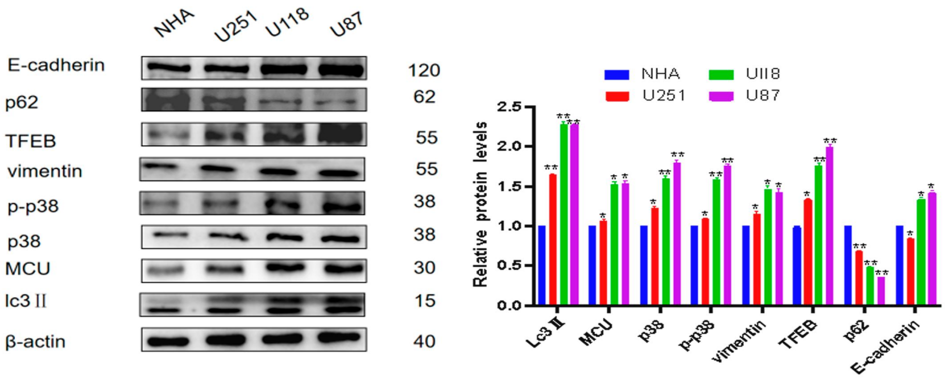

S1

A,B:Analysis of the protein expression changes of E-cadherin, p62, TFEB, vimentin, p-p38, p38, MCU, and lc3 II in NHA, U251, U118, U87 cell lines and the corresponding results,the results are expressed as mean ± SD (* P <0.05, * * P <0.01).

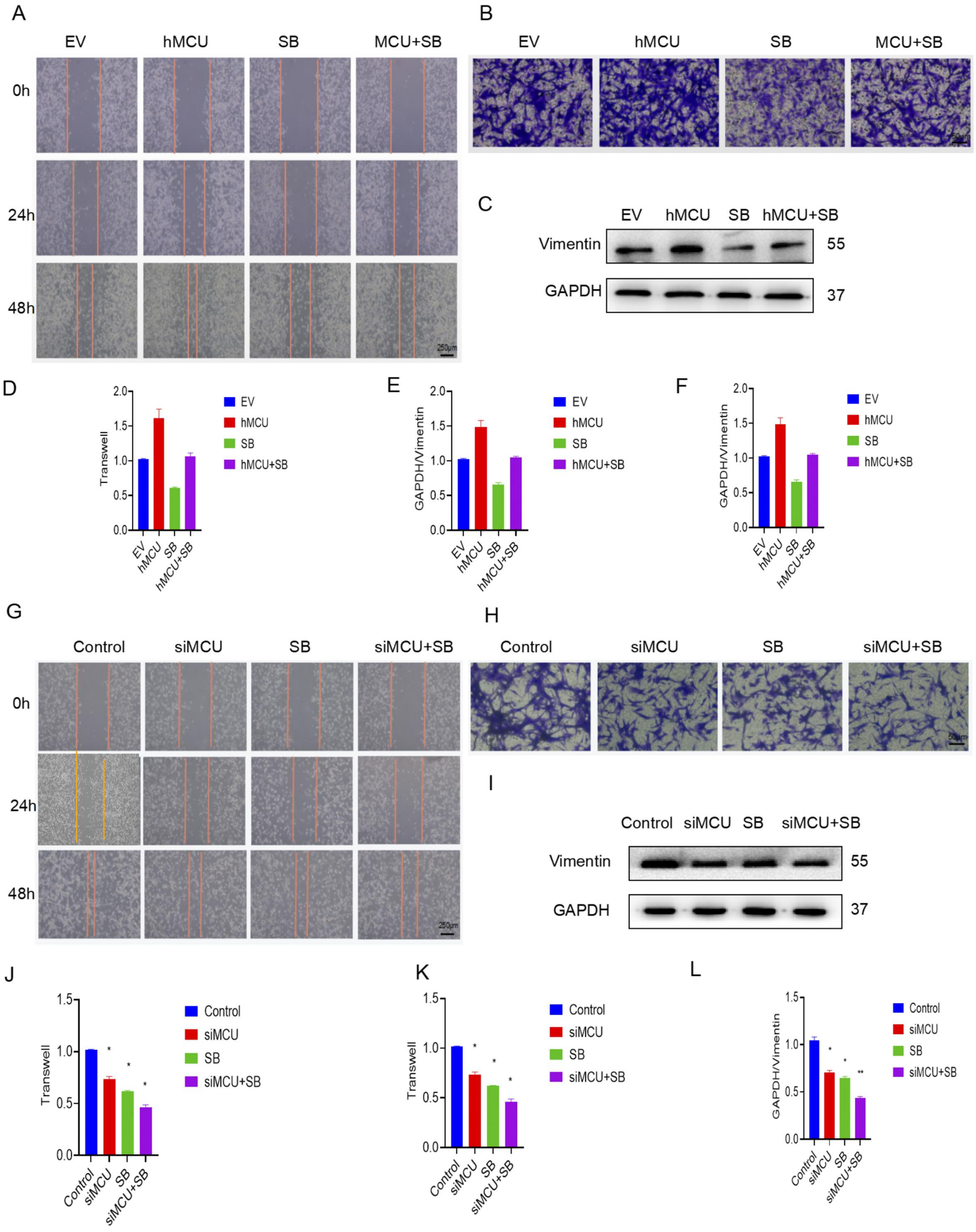

S2.

A, B, D, E): The VSVG-pLKD-U6-hMCU plasmid was transfected into U87 cells, and SB was added for cell scratch experiment (ruler 250μm) and

transewll experiment (ruler 50μm) to detect migration; C, F): WB detects protein Vimentin expression; G,H, J, K):The specific siRNA interference MCU plasmid was transfected into U87 cells and SB was added for cell scratch assay (ruler 250μm) and transewll experiment (ruler 50μm) to detect the migration; I,L): WB detected the expression of protein Vimentin,the results were expressed as mean ± SD.(* $P < 0.05$，** $P < 0.01$)

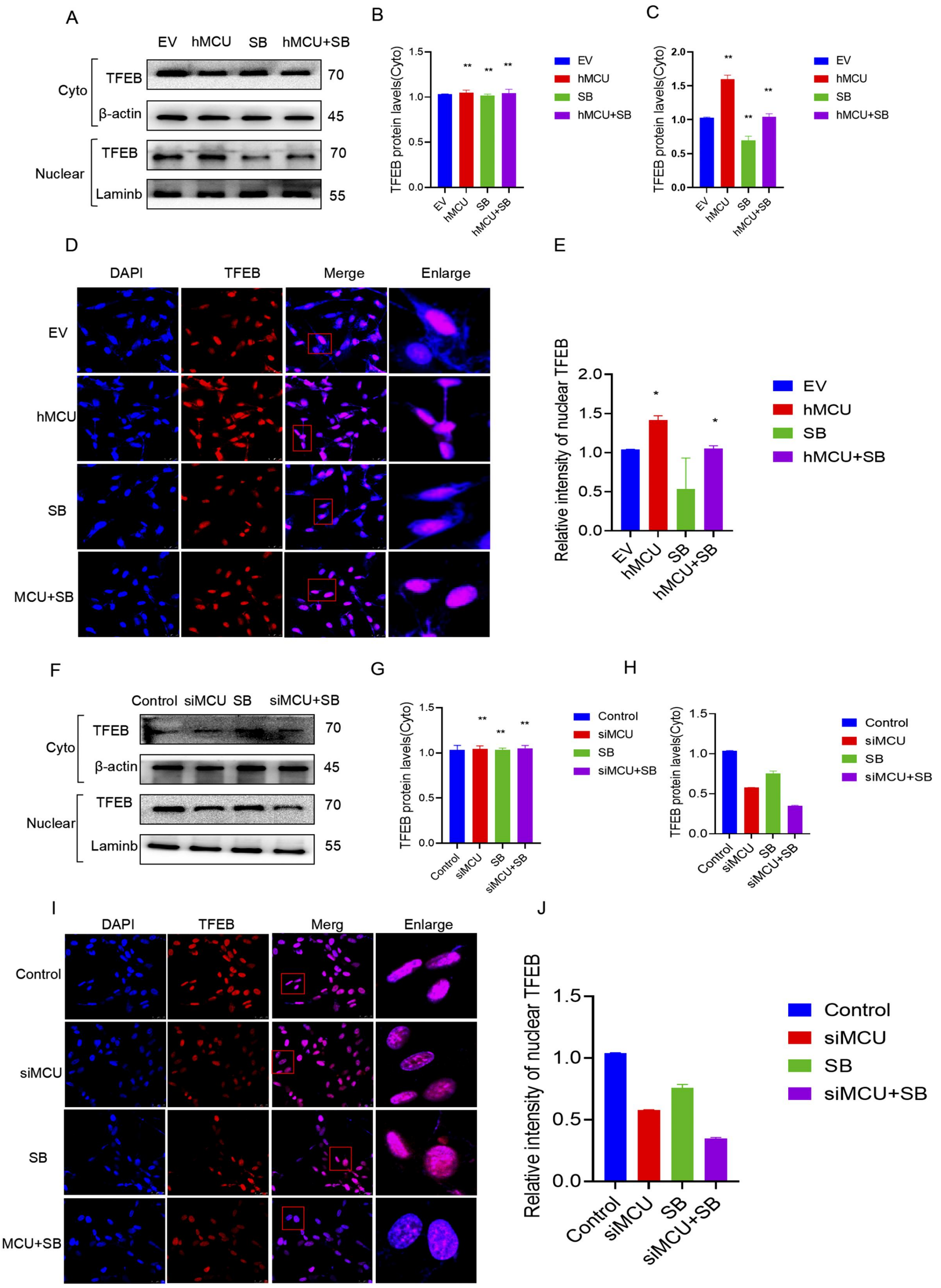

S3.

A-C):The hMCU was transfected into U87 cell, SB was added,WB detected TFEB levels in cytoplasm and nucleus;D,E): IF detected the nuclear translocation level of TFEB after adding SB;F-H):The siMCU was transfected into U87cell, SB was added;I-J): After addingSB, IF detected

the nuclear translocation level of TFEB;results are expressed as mean ± SD.(*$P < 0.05$，**$P < 0.01$)
